# The Intergenerational Transfer of Mental Disorders: A Population-based Multigenerational Linkage Study.

**DOI:** 10.23889/ijpds.v7i3.1874

**Published:** 2022-08-25

**Authors:** Amani Hamad, Barret Monchka, Leslie Roos, James Bolton, Oleguer Plana-Ripoll, Mohamed Elgendi, Lisa Lix

**Affiliations:** 1 University Of Melbourne; 2 Aarhus University; 3 Biomedical and Mobile Health Technology Lab

## Objectives

Mental disorders are a major public health concern. Genetic and environmental factors, both reflected in family health histories, jointly contribute to the onset of mental disorders. We examined the intergenerational transmission of mental disorders using objectively-measured family health histories from three generations.

## Approach

A population-based cohort study was conducted using administrative healthcare databases from Manitoba, Canada. The cohort included offspring who were 18 years or older between 1977 and 2020 with linkage to 1+ parent and 1+ grandparent. Mental disorders were identified using diagnosis codes from hospitalization and outpatient physician visit records and included mood and anxiety, psychotic, and substance use disorders. Logistic regression models were mutually adjusted for mental disorder history in grandparents, parents and/or siblings in addition to offspring demographics: sex, region, decade of birth and income quintile, and comorbidity. Odds ratios (ORs) and 95% confidence intervals (95% CIs) were estimated.

## Results

Out of 125,070 individuals, 59.1% were females and 57.8% were urban residents. 41,552 (33.2%) had a mental disorder during study period and 108,682 (86.9%) had a family member with a mental disorder history. Individuals were more likely to have a mental disorder if they had a family history: mother (OR 1.52, 95% CI 1.48-1.56), father (OR 1.21, 95% CI 1.17-1.25), sibling (OR 1.33, 95% CI 1.28-1.39), grandparent (OR 1.06, 95% CI 1.03-1.09). Compared with other mental disorders, psychotic disorders had the strongest association with family history: mother (OR 2.37, 95% CI 2.00-2.82), father (OR 3.00, 95% CI 2.40-3.76), sibling (OR 2.34, 95% CI 1.79-3.05). However, there was no association between psychotic disorders and grandparent history (OR 1.00, 95% CI 0.90-1.11).

## Conclusion and Relevance

We observed a strong association between mental disorders family history across three generations and the risk of the mental disorders in offspring. This association was observed for all the investigated mental disorders. This work highlights the value of multigenerational data linkage in understanding the intergenerational transfer of mental disorders.

